# A randomized controlled trial investigating the effect of liraglutide on self-reported liking and neural responses to food stimuli in participants with obesity

**DOI:** 10.1038/s41366-023-01370-w

**Published:** 2023-08-25

**Authors:** Géraldine Coppin, David Muñoz Tord, Eva R. Pool, Loïc Locatelli, Amal Achaibou, Asli Erdemli, Laura León Pérez, Lavinia Wuensch, Donato Cereghetti, Alain Golay, David Sander, Zoltan Pataky

**Affiliations:** 1 Swiss Center for Affective Sciences, University of Geneva, Geneva, Switzerland; 2 Department of Psychology, University of Geneva, Geneva, Switzerland; 3 Department of Psychology, UniDistance Suisse, Brig, Switzerland; 4 Division of endocrinology, diabetes, nutrition and therapeutic patient education, WHO Collaborating Centre, Geneva University Hospitals and University of Geneva, Geneva, Switzerland

**Keywords:** Obesity, Cognitive neuroscience

## Abstract

**Background/Objectives:**

Obesity is a complex condition and the mechanisms involved in weight gain and loss are not fully understood. Liraglutide, a GLP-1 receptor agonist, has been demonstrated to successfully promote weight loss in patients with obesity (OB). Yet, it is unclear whether the observed weight loss is driven by an alteration of food liking. Here we investigated the effects of liraglutide on food liking and the cerebral correlates of liking in OB.

**Subjects/Methods:**

This study was a randomized, single-center, double-blind, placebo-controlled, parallel group, prospective clinical trial. 73 participants with OB and without diabetes following a multidisciplinary weight loss program, were randomly assigned (1:1) to receive liraglutide 3.0 mg (37.40 ± 11.18 years old, BMI = 35.89 ± 3.01 kg) or a placebo (40.04 ± 14.10 years old, BMI = 34.88 ± 2.87 kg) subcutaneously once daily for 16 weeks.

**Interventions/Methods:**

We investigated liking during food consumption. Participants reported their hedonic experience while consuming a high-calorie food (milkshake) and a tasteless solution. The solutions were administered inside the scanner with a Magnetic Resonance Imaging (MRI)-compatible gustometer to assess neural responses during consumption. The same procedure was repeated during the pre- and post-intervention sessions.

**Results:**

None of the effects involving the intervention factor reached significance when comparing liking between the pre- and post-intervention sessions or groups. Liking during food reward consumption was associated with the activation of the ventromedial prefrontal cortex (vmPFC) and the amygdala. The liraglutide group lost more weight (BMI post-pre = −3.19 ± 1.28 kg/m^2^) than the placebo group (BMI post-pre = −0.60 ± 1.26 kg/m^2^).

**Conclusions:**

These results suggest that liraglutide leads to weight loss without self-report or neural evidence supporting a concomitant reduction of food liking in participants with OB.

## Introduction

Obesity is a complex condition associated with cognitive, affective, behavioral, cerebral, and metabolic dysfunctions [1–5]. The mechanisms involved in weight gain and weight loss are being actively researched and pharmacologic treatments of overweight and obesity have significantly evolved over the past years.

Liraglutide, a glucagon–like peptide 1 (GLP-1) receptor agonist, has been demonstrated to successfully promote weight loss. GLP-1 analogs were initially used in the treatment of patients with type 2 diabetes for their glucose–lowering effects. They have shown to have a beneficial impact on weight loss by exerting an anorectic effect [6]. They are now used for their weight loss effects in patients with obesity (OB). And yet, as of today, the exact mechanisms of GLP-1 agonists in weight loss are not fully understood. The first and most obvious hypothesis is that GLP-1 agonist-related side effects (e.g., nausea, vomiting) are responsible for a decrease in appetite and weight reduction; however, most treated patients lose weight in the absence of clinically significant side effects [7, 8]. Now if we investigate this question a little further and examine brain mechanisms, one could speculate that since GLP-1 receptors are located in areas linked to reward processing [9] (e.g., the orbitofrontal cortex), then GLP-1 agonists could affect reward processing (e.g., [9, 10]). Decades of research in affective neuroscience have shown that reward processing can be parsed into motivational and hedonic components [11–13]. The motivational component is defined as the motivation to obtain a reward (i.e., wanting); the hedonic component encompasses the subjective hedonic experience (i.e., liking) [14]. Wanting and liking also differ in their neurobiological substrates as they rely on different mesocorticolimbic circuits [11]. A prevalent idea of what drives our reward-seeking behavior assumes a hedonic perspective: what we want directly depends on what we like. While these two states are usually in synchronization, they can also dissociate under certain circumstances. For instance, people with addiction disorders frequently report an irrational wanting of a drug despite not liking its effects anymore [15]. A key observation in the investigation of the shift from voluntary consumption towards compulsive consumption is the increasing gap between wanting and liking, which is formally conceptualized in the incentive-sensitization theory of addiction (IST) [16].

What do we know about the effects of GLP-1 receptor agonists on wanting and liking?

There is evidence that GLP-1 receptor agonists affect food reward responses in patients with OB (e.g., [17]). For instance, it has been shown that an intravenous injection of exenatide, a GLP-1 receptor agonist, decreased anticipatory responses to the consumption of a chocolate milkshake. In a similar vein, liraglutide has been found to exert central effects by decreasing attention to highly desirable food cues, as shown by decreased parietal cortex activation [18]. Additionally, 17 days of chronic GLP-1 analog treatment decreased brain responses (e.g., in the insular cortex) to palatable food cues [19]. Farr et al. [20], however, showed that liraglutide increases the right orbitofrontal cortex’s response to food cues when administrated long-term (5 weeks of chronic GLP-1 analog treatment) at a high dose. More recently, Hanssen et al. [21] have shown behavioral evidence of GLP-1 modulation of incentive motivation on food rewards but did not investigate the neural correlates of this effect. This study suggests that wanting could be modulated by GLP-1 analogs.

Schulz et al. [22] recently pointed out that most studies in the field so far have focused on reward anticipation. However, flavor preferences can also be altered by chronic GLP-1 analog treatment [23], in women in particular [24]. Thus, although more research assessing the relationship between GLP-1 analogs and liking is needed, these studies suggest a potential effect of liraglutide on liking [25]. Unfortunately, fMRI studies examining the effects of GLP-1 receptor agonists on reward circuitry in patients with OB and using food stimuli administered by an fMRI-compatible gustometer are limited. One notable exception is the study conducted by Van Bloemendaal et al. [17]. These authors have shown that exenatide increases brain activity (e.g., in the left insula, left amygdala, and bilateral putamen) to the consumption of a chocolate milkshake. The participants were not asked to rate how much they liked the milkshake. We consequently believe investigating the effects of liraglutide on food liking and the cerebral correlates of liking in participants with OB using rewarding food stimuli delivered in the scanner is an important gap to fill.

Here we followed Schulz et al.’s [22] recommendation to consider other reward phases than anticipation and we focused on the liking component of reward. More specifically, we investigated whether liraglutide affects food liking in participants with OB without diabetes. Recent work has shown decreased food liking after weight loss [26, 27]. For instance, Oustric et al. [26] showed no change in wanting but a change in liking after a weight loss superior to 5%. This result suggests a potential link between food liking and weight loss. Given that liraglutide typically induces a similar weight loss [28–30], we hypothesized that liraglutide would decrease food liking. Importantly, this hypothesis should be considered with caution given that the causal relationships between food liking and weight loss are not clear, and because the specific action mechanisms of liraglutide on both food liking and weight loss are still to be understood. Our participants with OB without diabetes were separated into an intervention group receiving daily liraglutide injections and a control group receiving daily placebo injections over 16 weeks. In these two groups of participants, we used self-reports and measured brain activity to compare their response to a milkshake versus a tasteless solution.

To measure liking, we targeted the sensory pleasure during the consumption of the reward itself. As evidenced by Pool et al’s review [12], liking and wanting are typically investigated in the animal literature. In animals, the liking component of reward is measured during the reward consumption through orofacial reactions. Since our study was conducted in humans, we thought it was important to use experimental tasks that reflect the same processes as in animals and to adopt a similar operationalization. We do not exclude that a food cue could trigger a sensory pleasure experience after strong and consistent associative learning; in terms of mechanisms, we believe we are much closer to the construct of liking reported in the animal literature when measuring the sensory pleasure experience during the reward consumption.

## Materials and methods

### Trial overview

**Fig. 1:**
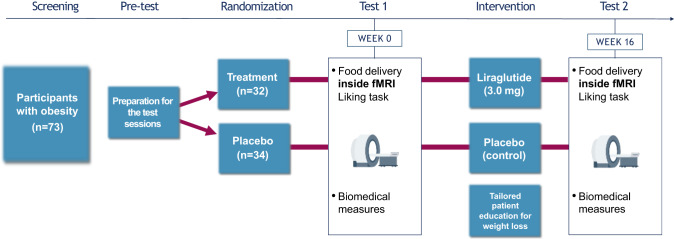
Trial overview. Participants with obesity participated in a pre-intervention session before being randomly assigned to the treatment (liraglutide) or placebo (control) group. During this first test session, they performed a liking task in the scanner. Biomedical measures were also taken, and they performed other experimental tasks not described here. After 16 weeks of treatment, they came back to the laboratory and went through a second test session.

The data reported here were acquired in the context of a larger study, components of which have been [31] and will be reported separately. More precisely, we conducted this randomized, single-center, double-blind, placebo-controlled, parallel group, prospective clinical trial from March 7, 2018 through March 18, 2020 at the Geneva University Hospitals (Switzerland). Since this clinical trial was conducted before the COVID-19 pandemic, we can reasonably exclude COVID-19 loss of smell and taste in the recruited patients. The trial was carried out in accordance with the protocol and principles enunciated in the current version of the Declaration of Helsinki, the guidelines of Good Clinical Practice (GCP) issued by ICH, and the Swiss Law and Swiss regulatory authority’s requirements. The trial was registered on ClinicalTrials.gov (NCT03347890) and the protocol as well as any subsequent amendments were approved by the local ethical committee (Commission Cantonale d’Ethique de la Recherche, CCER, Genève) and by the Swiss Agency for Therapeutic Products (Swissmedic). Participant safety was monitored from the screening to week 16 by an independent data monitoring committee. Participants were informed of the aims of the study and gave their written consent before any trial-related procedures. The trial overview is presented in Fig. 1.

### Participants

#### Multidisciplinary weight loss program

Participants with obesity (Body Mass Index, BMI ≥ 30 kg/m^2^ and < 45 kg/m^2^) were recruited from the Endocrinology, Diabetology, Nutrition, and Therapeutic Patient Education Division at the Geneva University Hospitals. They were part of a structured and multidisciplinary patient educational weight loss program. This weight loss program, based on lifestyle counseling (combining a group and an individual approach), included cognitive behavioral therapy coupled with a diet and physical activity support (described in detail in Pataky et al. [32]). Patients attended individual and group counseling sessions during the 16-week period; they were delivered by qualified health care professionals (registered dieticians, nurses, and physicians specialized in obesity treatment and patient education). The weight loss program was consequently tailored to each patient’s needs. All participants gave written informed consent and received 200 Swiss Francs (the equivalent of 200 USD) for their participation in the pre- and post-intervention sessions.

#### Inclusion and exclusion criteria

The inclusion criteria were as follows: between 18 and 75 years old, stable body weight (<5% reported change within 3 months before the screening), right-handedness, and currently a non-smoker. The exclusion criteria were based on contraindications to liraglutide treatment, any drugs interfering with body weight control (e.g., Orlistat, Phentermine and Topiramate, Buproprion and Naltrexone) or any centrally acting medication, any history of psychiatric disease, heart failure (NYHA II-IV), type 1 and type 2 diabetes mellitus, and deficits of smell and taste. The complete list of eligibility criteria is listed in the supplementary information.

#### Trial population

A total of 73 participants with OB were screened. After determining their study eligibility, 66 participants were included in the trial. Those participants were randomly assigned to one of two groups: 32 received liraglutide 3.0 mg combined with lifestyle counseling and 34 received a placebo combined with lifestyle counseling (see Fig. 1 in Supplementary Information). Baseline characteristics of the studied population are described in Table 1 (see Supplementary Information). In total, 22 participants were excluded from the analysis (10 participants did not complete the second testing session and 12 participants had missing or corrupt MRI data). We report data on the 44 remaining participants (liraglutide group: age 37.4 years ± 11.18, BMI 35.89 kg/m^2^ ± 3.01, *n* = 20; placebo group: age 40.04 years ± 14.1, BMI 34.88 ± 2.87 kg/m^2^, *n* = 24).

**Table 1:** Summary results of BOLD activations for the liking main effect.

Cerebellum	L	1	19.406	−10	−69	−61
Cerebellum	R	1	17.576	12	−60	−54
White matter	L	13	27.034	−16	−51	−36
Middle temporal gyrus	L	11	31.545	−67	−21	−21
Amygdala	L	50	37.946	−22	−6	−18
Subcallosal cortex	L	50	32.184	−10	7	−14
Amygdala	R	4	23.287	15	−6	−18
Amygdala	R	2	18.802	24	−3	−18
Not in atlas	L	1	17.914	−1	−3	−18
Ventromedial PFC	R	3	19.947	3	49	−18
Ventromedial PFC	L	4	19.991	−7	34	−14
Putamen	R	1	17.609	33	−12	4
Frontal pole	R	22	25.258	9	67	15
Frontal pole	L	3	17.674	−7	64	11
Lateral occipital cortex	R	2	18.192	39	−75	22
White matter	R	2	18.308	15	19	26
Lateral occipital cortex	R	56	29.572	12	−75	62
Lateral occipital cortex	L	2	20.321	−16	−78	62

Thresholding *p* < 0.001 voxel wise FDR corrected. Table shows the peak level statistics and coordinates. Coordinates are expressed in the Montreal Neurological Institute (MNI) space in the left-right, anterior–posterior, and inferior-superior dimensions, respectively.

We ran a sensitivity analysis on the equivalent ANOVA model to the linear mixed model we report below, testing the 2 taste stimuli × 2 interventions × 2 pre–post effects on the liking ratings. This analysis revealed that this sample size allowed for the detection of a smallest population effect size of 0.177 (Cohen’s *f*), which is interpreted as a small to medium effect. Concretely, this means that our study, given its design and its sample, was able to detect a small to medium effect of the intervention on liking perception.

### Taste stimuli and presentation

We used two types of stimuli in this experiment: (1) a rewarding stimulus consisting of a chocolate milkshake solution and (2) a control stimulus consisting of a tasteless solution.

We prepared the milkshake by mixing a chocolate flavored milk drink (300 g) with a Fior Di Latte flavored ice cream (60 g) for a total of 71 kcal/100 g.

The tasteless solution, which was meant to be as close as possible to artificial saliva, was prepared in three steps. First, we diluted potassium chloride (KCl, 1.8 g) and sodium bicarbonate (NaHCO_3,_ 0.21 g) in 1 L of distilled water. Second, we created three less concentrated versions of this solution; thus, there were 4 different tasteless concentrated solutions (1/1, ¾, ½, and ¼). Third, participants were invited to taste the 4 solutions. The one that tasted the most neutral to them (i.e., closest to 50 on a scale from 0 “very unpleasant” to 100 “very pleasant”) was used as their tasteless solution. We believe using one of these 4 tasteless solutions was more suitable as the control stimulus than using water because water has an inherent taste [33].

The milkshake and the tasteless solution were kept in the fridge. We took them out simultaneously 30 minutes before the experiment so that they were delivered at ambient temperature.

The apparatus used to deliver the liquids in the scanner has been described in Muñoz-Tord et al. [31]. In a nutshell, a 3D-printed pacifier-shaped fMRI mouthpiece paired with a gustometer was used to deliver the liquids while participants were lying down. As demonstrated in Muñoz-Tord et al., this allows for a precise, reliable, and comfortable liquid delivery method. Data collection was controlled by a computer running MATLAB (version R2015a; MathWorks, Natick, USA). The presentation of the visual stimuli was implemented using Psychtoolbox (version 3.0) [34].

### Procedures

Participants who fulfilled the study criteria were randomly assigned in a 1:1 ratio to receive liraglutide 3.0 mg or a placebo, which was administered subcutaneously using pen injectors. After a dose escalation period starting at 0.6 mg q.d., there were weekly increments of 0.6 mg (see Fig. 2 in Supplementary Information). The placebo pen injector was identical to the liraglutide pen injector. A follow-up with the participants occurred on a weekly basis during the 5-week dose escalation period, and on a monthly basis afterwards.

### Metabolic measures

Blood samples for study purposes were collected both at baseline and at the 16-week follow-up. Fasting plasma blood glucose, insulin, plasma lipids, and HbA1c were measured in fasting conditions using routine biochemistry test.

### Experimental procedure

The experiment consisted of three separate testing days (see Fig. 1). On the first testing day, participants came to the laboratory for a pretest (see description below). On the second testing day, participants came for a test session before the beginning of the intervention (i.e., baseline testing session); this session took place in the morning. All participants were asked to fast overnight. Participants were requested to have eaten their last meal at least 8 h prior to the session. Afterwards, participants underwent the intervention (placebo + counseling vs liraglutide + counseling) and came back to the laboratory for the third testing day at the end of the intervention for the second test session (i.e., 16-week follow-up testing session). Both test sessions followed the exact same procedure.

Please note that during the test sessions, participants performed multiple experimental tasks; here, we only report the results for the hedonic reactivity task.

#### Pretest

Participants chose the most neutral tasteless solution to them. The 4 solutions were presented to them in shot glasses (=1 dl). Participants self-reported their current hunger level as well as pleasantness, intensity, and familiarity levels for their selected tasteless solution and for the milkshake (see Table 2, Supplementary Information). They also underwent 10–20 min of structural scans in the MRI. This small fMRI session allowed them to be more confident and comfortable for the longer functional scans taking place during the test sessions.

#### Test session

**Fig. 2:**
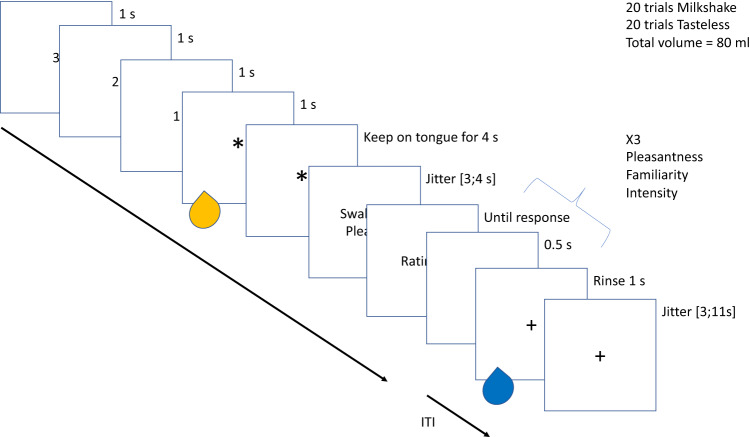
Overview of the liking task. This task was performed while participants were lying in the scanner and equipped with a 3D-printed pacifier-shaped fMRI mouthpiece paired with a gustometer. After a countdown (3–1) on a screen, participants saw a fixation cross followed by the delivery of a tasteless solution or a chocolate milkshake in their mouths. They were requested to keep the solution on their tongues for 4 s before being asked to swallow it. They were asked to rate the pleasantness, familiarity, and intensity of the solution. Rinse trials were intertwined with experimental trials so that participants could cleanse their palates.

We administered a liking task while participants were lying in the scanner. The task consisted in the evaluation of the perceived pleasantness, intensity, and familiarity of the two different stimuli (the milkshake and the tasteless solution). Participants were instructed to assess the solutions, focusing on their current perception of them. During each trial, 1 mL of the solution was administered, and the delivery order of the two conditions was randomized for each participant. Participants were visually guided through the task with on-screen instructions. First, they saw a 3-s countdown before the solution was delivered, followed by an asterisk presented for 4 s instructing them to keep the solution on their tongue until they saw the instruction to “swallow please” (see Fig. 2). We asked them to wait 4 s before swallowing to avoid motion artifacts in the Blood-Oxygen-Level-Dependent (BOLD) response. Since they were lying down, the mouthpiece was placed in such a way that the solution was delivered at the center of the participants’ tongues. We expected the solution would slide down to the back of their tongue over the 4-s period. The experimental trials were intertwined with rinse trials to cleanse the participants’ palates with 1 mL of water. All 40 evaluations (20 per solution) were done on visual analog scales displayed on a computer screen. Participants had to answer using a button-box placed in their hand. The visual analog scales ranged from “not perceived” to “extremely intense” for the intensity ratings; from “extremely unpleasant” to “extremely pleasant” for the liking ratings; and from “extremely familiar” to “extremely unfamiliar” for the familiarity ratings.

### Statistical analysis

#### Behavioral and metabolic data

We analyzed the behavioral and metabolic data with R (version 4.0; R Core Team, 2019).

We built two statistical models. The first model aimed at testing the relationship between weight loss (measured by subtracting the participants’ BMI after the intervention from their BMI before the intervention) and the intervention. We entered (1) intervention (placebo or liraglutide) as a fixed effect and (2) age and (3) sex as control factors. We entered intercepts for participants as a random effect. We built the model as follows:

$${\rm{Weight}}\,{\rm{loss}} \sim {\rm{intervention}}+{\rm{sex}}+{\rm{age}}+(1|{\rm{id}})$$

The second model aimed at testing the relationship between the perceived pleasantness of the tastes and the intervention. We entered (1) the taste stimulus (milkshake or tasteless), (2) session (pre- or post-intervention), (3) intervention (placebo or liraglutide), and (4) a linear decreasing contrast over trials to account for satiation as fixed factors. For the random effects, we entered intercepts for participants as well as by-participant random slopes for the effect of the interaction between taste stimulus sessions and trials. We did so to reduce the likelihood of a false positive. From a conceptual point of view, we expected that the intervention effect on the satiation processes for the milkshake would differ from one participant to another (e.g., the effect is large for some, medium for others, etc.) We built the model as follows:

$$\begin{array}{l}{\rm{Liking}} \sim {\rm{intervention}}\times{\rm{stimulus}}\times{\rm{session}}\times{\rm{satiation}}\\\qquad\quad+({\rm{stimulus}}\times{\rm{session}}\times{\rm{satiation}}|{\rm{id}})\end{array}$$

We used the lme4 package [35] and the LmerTest package [36]. We extracted Bayes factors through linear mixed Bayesian analysis using brms [37], CmdStanR [38] and bayestestR [39] packages. The models were estimated using Markov chain Monte Carlo (MCMC) sampling with 4 chains of 5000 iterations and a warmup of 1000. The dependent variables were scaled before being entered in the model. Prior parameters were set as normal distributions (*M* = 0.00, SD = 1.00) for the first model (weight loss) as well as the second model (perceived pleasantness). The Bayes factors reported for the main effects compared the model with the main effect in question versus the null model. Furthermore, the Bayes factors reported for the interaction effects compared the model including the interaction term with the model including all the other effects but the interaction term. Evidence in favor of the model of interest could be considered anecdotal (1 < BF_10_ < 3), substantial (3 < BF_10_ < 10), strong (10 < BF_10_ < 30), very strong (30 < BF_10_ < 100), or decisive (BF_10_ > 100). Similarly, evidence in favor of the null model could also be qualified as anecdotal (0.33 < BF_10_ < 1), substantial (0.1 < BF_10_ < 0.33), strong (0.033 < BF_10_ < 0.1), very strong (0.01 < BF_10_ < 0.033), or decisive (BF_10_ < 0.01).

### fMRI data

#### Acquisition parameters

Acquisition parameters were identical to the ones described in Muñoz-Tord et al. [31]. The neuroimaging data were acquired on a 3-Tesla MRI system (Magnetom Tim Trio, Siemens Medical Solutions) supplied with a 32-channel head coil using a gradient echo (GRE) sequence to record the BOLD signal. We recorded forty echo-planar imaging (EPI) slices per scan with an isotropic voxel size of 3 mm. The scanner parameters were set at: echo time (TE) = 20 ms, repetition time (TR) = 2000 ms, field of view (FOV) = 210 × 210 × 144 mm, matrix size = 70 × 70 voxels, flip angle = 85°, 0.6 mm gap between slices. Structural whole brain T1-weighted (T1w) images (isotropic voxel size = 1.0 mm) were acquired as well as dual gradient B0 field maps (Fmaps) for each participant to correct for inhomogeneity distortions in the static-field.

#### Preprocessing

We created a pipeline optimized for the preprocessing of our neuroimaging data identical to Muñoz-Tord et al.’s study [31]. More specifically, we combined the Functional Magnetic Resonance Imaging of the Brain (FMRIB) Software Library (FSL, version 4.1) [40] with the Advanced Normalization Tools (ANTS, version 2.1) [41]. The BOLD signal is highly prone to motion artifacts. This type of noise made our experimental setting particularly challenging to analyze since our participants swallowed solutions in the scanner, thereby producing substantial deglutition artifacts. To offset this loss of signal-to-noise ratio (SNR), we followed Griffanti et al.’s protocol [42]. This protocol uses an fMRI independent component analysis (ICA) to remove artifacts. The multivariate exploratory linear optimized decomposition tool (MELODIC) [43] decomposes the raw BOLD signal into independent components (IC). We chose this ICA-based strategy for motion artifact removal because it is more reliable to remove motion-induced signal variations than regressions from motion parameters [44]. Two researchers from our laboratory independently hand classified a sample of 20 participants’ IC into two categories: ‘potential signal’ or ‘clear artifact’ (e.g., motion/deglutition, blood flow in arteries). The two researchers’ categorizations were then compared, and each discrepancy was discussed until an agreement was reached (inter-rater reliability = 93%). This process allowed us to manually classify components. These components were then used to train a classifier using a random forest machine learning algorithm [45]. We used leave-one-out testing during which we iteratively left one participant out of the training data and tested the classifier’s accuracy on the left-out participant. Leave-one-out testing at the optimal sensitivity (threshold = 5) resulted in a median 94% true positive rate (i.e., the percentage of ‘true signal’ accurately classified). We consequently applied the FMRIB’s ICA-based X-noiseifier (FIX) to automatize the denoising of our BOLD signal [46]. We then applied field maps to correct geometric distortions. We used ANTS for a diffeomorphic co-registration of the preprocessed functional and structural images in the Montreal Neurological Institute (MNI) space; we used the nearest-neighbor interpolation and left the functional images in their native resolution. Finally, we applied a spatial smoothing of 8 mm full width at half maximum (FWHM).

#### Statistical analysis

We used the Statistical Parametric Mapping software (SPM, version 12) [47] to perform a random-effects univariate analysis on the voxels of the image times series using a two-level approach.

For the first-level, we specified a general linear model (GLM) for each participant. We used a high-pass filter cutoff of 1/128 Hz to eliminate possible low-frequency confounds. Each regressor of interest was derived from the onsets of the stimuli and convoluted using a canonical hemodynamic response function (HRF) into the GLM to obtain weighted parameter estimates. The GLM consisted of eight regressors: (1) the onset of the trial, (2) the onset of the reception of a taste stimulus modulated by (3) the presence of the milkshake, (4) the trial-by-trial ratings of the perceived pleasantness, (5) the onset of the pleasantness question, (7) the onset of the intensity question, and (8) the onset of the familiarity question. We extracted the taste delivery contrast modulated by the perceived pleasantness for each participant for each session (43 participants × 2 sessions = 86).

For the second-level, we entered the first level contrasts in a mixed measures 2 (session: pre or post) by 2 (treatment: placebo or liraglutide) ANOVA using the multivariate and repeated measures toolbox (MRM) [48]. The MRM toolbox is a MATLAB toolbox allowing us to perform mass multivariate group models of neuroimaging data using the summary statistic approach by selecting the correct error term [49]. We extracted *F* contrasts with a voxel-wise significance threshold set at *p* < 0.001, FDR corrected for multiple comparisons. For display purposes, we plotted non-masked and uncorrected statistical *p*-maps of our group results overlaid on a high-resolution template (CIT 168) in the MNI space.

## Results

**Fig. 3:**
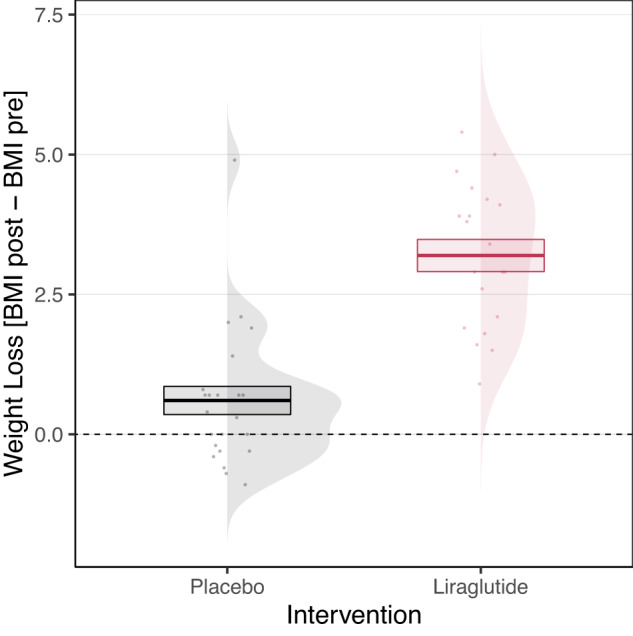
Weight loss results. Individual estimates, densities, and overall mean of the weight loss measured in BMI units (BMI post-intervention – BMI pre-intervention) of the placebo (*N* = 24) and liraglutide (*N* = 20) groups. Error bars represent standard errors of the mean.

Our first step was to establish whether we could replicate the well-documented liraglutide effect on weight loss. We thereby used a multilevel model to test the effect of the intervention on weight loss. A statistically significant effect of intervention was found (*β* = 0.704, *p* < 0.001; see Fig. 3 and Table 4 in the Supplementary Information), suggesting that liraglutide indeed led patients to lose more weight than patients receiving the placebo injections.

**Fig. 4:**
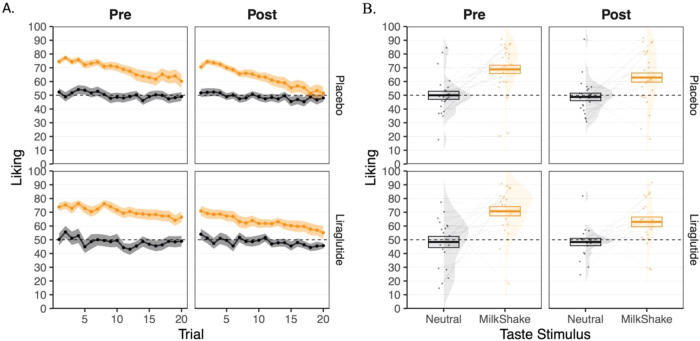
Liking for the rewarding and the neutral taste stimuli in the placebo (*N* = 20) and the liraglutide (*N* = 24) groups before (i.e., pre) and after (i.e., post) the intervention. **A** represents liking over trial repetition, while **B** represents individual estimates, densities, and overall liking mean. Error bars and shaded areas represent standard errors of the mean.

Next, we wanted to ascertain whether the intervention affected the self-reported liking of a rewarding taste (i.e., the milkshake). We thereby used a multilevel model testing the effect of the intervention on the reward’s liking. This model revealed a statistically significant effect of taste stimulus (*β* = −0.409, *p* < 0.001; see Fig. 4B and Table 5 in the Supplementary Information), a main effect of satiation (*β* = 0.013, *p* < 0.001; see Fig. 4A) and an interaction effect between satiation and taste stimulus (*β* = −0.007, *p* = 0.002; see Fig. 4A). These results suggest that the milkshake was more satiating than the control taste. Moreover, we found a statistically significant main effect of session (*β* = 0.087, *p* = 0.045) and an interaction between taste stimulus and session (*β* = −0.073, *p* = 0.042). This suggests that the taste stimuli were perceived as less pleasant during the second session. None of the effect terms involving the intervention factor reached significance. There was no statistically significant interaction between taste stimulus, session, and intervention (*β* = 0.017, *p* = 0.617), nor between taste stimulus, session, satiation, and intervention (*β* < 0.001, *p* = 0.529). Thus, we did not find any statistical evidence that the intervention impacted self-reported liking.

**Fig. 5:**
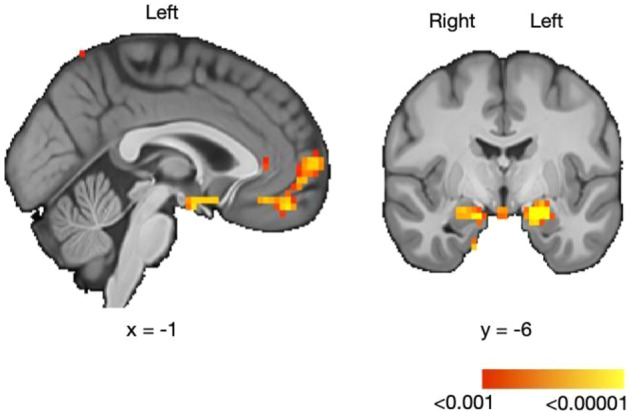
Neuronal correlates of liking. Regions where the BOLD signal positively correlated with the magnitude of the liking experienced within participants (*N* = 44). For display purposes, statistical maps are shown with a threshold of 0.001 uncorrected. Color scale bar represents *p* values. Detailed results are presented in Table 1.

Regarding the fMRI data, we tested the interaction between the intervention (placebo or liraglutide) × the session (pre- or post-intervention) using a Repeated Measures ANOVA. There was no statistically significant voxel that survived FDR correction. In other words, there was no activation specific to a post- compared to pre-intervention change in the liraglutide group compared to the placebo group. However, the analysis revealed a main effect of the pleasantness modulator, which activated brain regions typically involved in reward processing such as the ventromedial prefrontal cortex (vmPFC; peak voxel coordinates : *x* = −7, *y* = 34, *z* = −14, *k* = 4; right peak voxel coordinates: *x* = 3, *y* = 49, *z* = −18; *k* = 3) and bilateral amygdala (left peak voxel coordinates: *x* = −22, *y* = −6, *z* = −18, *k* = 50; right peak voxel coordinates *x* = 24, *y* = −3, *z* = −18; see Fig. 5). Thus, amygdala and vmPFC activations correlated with perceived liking during taste consumption for both groups. A summary of the BOLD activations for the main effect of liking are displayed in Table 1.

## Discussion

In this randomized, single-center, double-blind, placebo-controlled, parallel group, prospective clinical trial, we investigated whether GLP-1 analog liraglutide affects food-related liking in participants with OB using an fMRI-compatible gustometer. We measured both self-reports of liking and neural responses.

Behaviorally, and contrary to our hypothesis, we did not find any statistical evidence that our intervention impacted food liking. We must always be careful when interpreting the absence of significant main effects or of a significant interaction effect. As in any experiment, we cannot exclude classical limitations such as methodological aspects or sample size. For instance, our sensitivity analysis showed that we could detect a small to medium effect of the intervention on liking perception, therefore we cannot exclude the presence of a small effect. However, our Bayesian approach provides strong evidence favoring the null hypothesis, which suggests that self-reported liking did not differ between the liraglutide and placebo groups or between pre- and post-intervention ratings.

As for the brain activity, analyses of the participants’ neural responses to the milkshake and control taste stimulus revealed a main effect of the liking modulator activating the vmPFC and the amygdala; these brain regions are known to be involved in reward processing [50, 51]. More specifically, valence-specific signals in the amygdala have been identified to modulate food choices [52] and the vmPFC has been found to be involved in decision-making about reward value [53]. Our fMRI resolution does not allow us to differentiate sub-regions of the amygdala or the vmPFC. However, different sub-areas have been identified to underlie different aspects of reward learning and food decision-making (e.g., [54]). Furthermore, the amygdala and the vmPFC are strongly connected [55] and a value-related connectivity between these two regions has been reported in previous studies (e.g., [56]). Again, while drawing conclusions from the absence of a significant interaction effect is not warranted, the results from our Bayesian approach are consistent with the proposal that there is no effect of the liraglutide treatment on liking. Additionally, we found that amygdala and vmPFC activations correlated with perceived liking during taste consumption for both groups.

Taken together, our results do not provide evidence that liraglutide alters liking while consuming food. This contrasts with recent findings showing that weight loss was associated with a decrease in food liking [26, 27]. It also differs from what is reported in the literature about the potential nauseating effects of liraglutide [57, 58], which could possibly spoil the pleasure of eating for patients experiencing this side effect. Indeed, we did not find liraglutide to affect the pleasant experience of consuming rewarding food. This is consistent with a recent review on the role of GLP-1 in humans, which did not find conclusive evidence of this hormone reducing reward responses [22].

While we did not find evidence suggesting that liraglutide changed the milkshake liking, we did find evidence indicating that the milkshake was less pleasant in the second session compared to the first for both groups. This effect was therefore independent of the GLP-1 analog treatment. It is unclear which factor(s) this effect is driven by given the absence of a control group without a weight loss intervention in our current study. It could be driven by the weight loss intervention, but differences in BMI did not significantly correlate with liking of the milkshake in a previous study using similar stimuli [59]. Another possibility is that it was driven by repetition or habituation to the milkshake, which are well documented effects (e.g., [60]).

Another well documented effect we found is a large effect of the medication on weight loss. As expected and previously documented [28–30], the liraglutide group significantly lost more weight than the placebo group. On average, the liraglutide group lost 8.96% (SD = 3.78) of their body weight, while the placebo group lost 1.67%, (SD = 3.38). This weight loss was comparable to previous studies [28–30].

However, the weight loss induced by liraglutide was not our main research question—the present study focused on the liking component of reward. While there are a few studies on the wanting component (e.g., [21]), research on the effects of GLP-1 on liking in humans with OB using food stimuli is rare. It is thereby difficult to compare our findings to previous studies while taking into consideration the treatment duration of GLP-1 analogs or the different GLP-1 analogs.

To conclude, this study suggests that liraglutide leads to weight loss but does not provide evidence that it is due to a change in food-related liking in participants with obesity.

### Supplementary information

Supplementary information

## Data Availability

The computer code used to preprocess and analyze the data is available in a publicly hosted software repository (for preprocessing of the fMRI data: https://github.com/munoztd0/Mouthpiecegusto/tree/main/preprocessing; for data analysis: https://github.com/evapool/GLP1_Pleasure).

## References

[1] Fitzpatrick S, Gilbert S, Serpell L (2013). Systematic review: are overweight and obese individuals impaired on behavioural tasks of executive functioning?. Neuropsychol Rev.

[2] Castanon N, Luheshi G, Laye S (2015). Role of neuroinflammation in the emotional and cognitive alterations displayed by animal models of obesity. Front Neurosci.

[3] Quercioli A, Montecucco F, Pataky Z, Thomas A, Ambrosio G, Staub C (2013). Improvement in coronary circulatory function in morbidly obese individuals after gastric bypass-induced weight loss: relation to alterations in endocannabinoids and adipocytokines. Eur Heart J.

[4] Nederkoorn C, Houben K, Hofmann W, Roefs A, Jansen A (2010). Control yourself or just eat what you like? Weight gain over a year is predicted by an interactive effect of response inhibition and implicit preference for snack foods. Health Psychol.

[5] Burger KS, Shearrer GE, Sanders AJ (2015). Brain-based etiology of weight regulation. Curr Diab Rep.

[6] Vilsbøll T, Zdravkovic M, Le-Thi T, Krarup T, Schmitz O, Courrèges JP (2007). Liraglutide, a long-acting human glucagon-like peptide-1 analog, given as monother- apy significantly improves glycemic control and lowers body weight with- out risk of hypoglycemia in patients with type 2 diabetes. Diabetes Care.

[7] Filippatos TD, Panagiotopoulos TV, Elisaf MS (2014). Adverse effects of GLP-1 receptor agonists. Rev Diab Stud.

[8] Gorgojo-Martínez JJ, Mezquita-Raya P, Carretero-Gómez J, Castro A, Cebrián-Cuenca A, de Torres-Sánchez A (2023). Clinical recommendations to manage gastrointestinal adverse events in patients treated with Glp-1 receptor agonists: A multidisciplinary expert consensus. J Clin Med.

[9] Eren-Yazicioglu CY, Yigit A, Dogruoz RE, Yapici-Eser H (2021). Can GLP-1 be a target for reward system related disorders? A qualitative synthesis and systematic review analysis of studies on palatable food, drugs of abuse, and alcohol. Front Behav Neurosci.

[10] van Bloemendaal L, Ijzerman RG, ten Kulve JS, Barkhof F, Konrad RJ, Drent ML (2014). GLP-1 receptor activation modulates appetite- and reward-related brain areas in humans. Diabetes..

[11] Berridge KC (2009). “Liking” and “wanting” food rewards: Brain substrates and roles in eating disorders. Physiol Behav.

[12] Pool EP, Sennwald V, Delplanque S, Brosch T, Sander D (2016). Measuring wanting and liking from animals to humans: a systematic review. Neurosci Biobehav Rev.

[13] Pool ER, Munoz Tord D, Delplanque S, Stussi Y, Cereghetti D, Vuilleumier P (2022). Differential contributions of ventral striatum subregions to the motivational and hedonic components of the affective processing of the reward. J Neurosci.

[14] Berridge KC, Robinson TE (2003). Parsing reward. Trends Neurosci.

[15] Hobbs M, Remington B, Glautier S (2005). Dissociation of wanting and liking for alcohol in humans: a test of the incentive-sensitisation theory. Psychopharmacology..

[16] Robinson TE, Berridge KC (1993). The neural basis of drug craving: an incentive-sensitization theory of addiction. Brain Res Rev.

[17] van Bloemendaal L, Veltman DJ, ten Kulve JS, Groot PFC, Ruhé HG, Barkhof F (2015). Brain reward-system activation in response to anticipation and consumption of palatable food is altered by glucagon-like peptide-1 receptor activation in humans. Diabetes Obes Metab.

[18] Farr OM, Sofopoulos M, Tsoukas MA, Dincer F, Thakkar B, Sahin- Efe A (2016). GLP-1 receptors exist in the parietal cortex, hypothalamus and medulla of human brains and the GLP-1 analogue liraglutide alters brain activity related to highly desirable food cues in individuals with diabetes: a crossover, randomized, placebo-controlled trial. Diabetologia..

[19] Farr OM, Tsoukas MA, Triantafyllou G, Dincer F, Filippaios A, Ko BJ (2016). Short-term administration of the GLP-1 analog liraglutide decreases circulating leptin and increases GIP levels and these changes are associated with alterations in CNS responses to food cues: a randomized, placebo-controlled, crossover study. Metabolism..

[20] Farr OM, Upadhyay J, Rutagengwa C, DiPrisco B, Ranta Z, Adra A (2019). Longer-term liraglutide administration at the highest dose approved for obesity increases reward-related orbitofrontal cortex activation in response to food cues: Implications for plateauing weight loss in response to anti-obesity therapies. Diabetes Obes Metab..

[21] Hanssen R, Kretschmer AC, Rigoux L, Albus K, Thanarajah SE, Sitnikow T (2021). GLP-1 and hunger modulate incentive motivation depending on insulin sensitivity in humans. Mol Metab.

[22] Schulz C, Vezzani C, Kroemer NB. How gut hormones shape reward: a systematic review of the role of ghrelin and GLP-1 in human fMRI. BioRxiv preprint. 2022. 10.1101/2022.11.30.518539. 10.1016/j.physbeh.2023.114111 36740132

[23] Kadouh H, Chedid V, Halawi H, Burton DD, Clark MM, Khemani D (2020). GLP-1 analog modulates appetite, taste preference, gut hormones, and regional body fat stores in adults with obesity. J Clin Endocrinol Metab.

[24] Baretić M, Kusec V, Uroić V, Pavlić-Renar I, Altabas V (2019). Glucagon-like peptide-1 affects taste perception differently in women: A randomized, placebo-controlled crossover study. Acta Clin Croat.

[25] Decarie-Spain L, Kanoski SE (2021). Ghrelin and glucagon-like peptide-1: a gut-brain axis battle for food reward. Nutrients..

[26] Oustric P, Beaulieu K, Casanova N, O’Connor D, Gibbons C, Hopkins M (2021). Food liking but not wanting decreases after controlled intermittent or continuous energy restriction to ≥ 5% weight loss in woman with overweight/obesity. Nutrients..

[27] Aukan MI, Brandsæter IØ, Skårvold S, Finlayson G, Nymo S, Coutinho S (2022). Changes in hedonic hunger and food reward after a similar weight loss induced by a very low-energy diet or bariatric surgery. Obesity..

[28] Davies MJ, Bergenstal R, Bode B, Kushner RF, Lewin A, Skjøth TV (2015). Efficacy of liraglutide for weight loss among patients with type 2 diabetes: The SCALE diabetes randomized clinical trial. JAMA..

[29] Pi-Sunyer X, Astrup A, Fujioka K, Greenway F, Halpern A, Krempf M (2015). for the SCALE Obesity and Prediabetes NN8022-1839 Study Group. A randomized, controlled trial of 3.0 mg of Liraglutide in weight management. N Engl J Med.

[30] Wadden TA, Tronieri JS, Sugimoto D, Lund MT, Auerbach P, Jensen C (2020). Liraglutide 3.0 mg and Intensive Behavioral Therapy (IBT) for obesity in primary care: The SCALE IBT randomized controlled trial. Obesity..

[31] Muñoz-Tord D, Coppin G, Pool E, Mermoud C, Pataky Z, Sander D, et al. 3D printed pacifier-shaped mouthpiece for fMRI-compatible gustometers. eNeuro. 2021.; 10.1523/ENEURO.0208-21.2021. 10.1523/ENEURO.0208-21.2021 PMC8496206 34551958

[32] Pataky Z, Carrard I, Gay V, Thomas A, Carpentier A, Bobbioni-Harsch E (2018). Effects of a weight loss program on metabolic syndrome, eating disorders and psychological outcomes: Mediation by endocannabinoids?. Obes Facts.

[33] Bartoshuk LM, McBurney DH, Pfaffmann C (1964). Taste of sodium chloride solutions after adaptation to sodium chloride: Implications for the “water taste”. Science..

[34] Brainard DH (1997). The psychophysics toolbox. Spat Vis.

[35] Bates D, Mächler M, Bolker B, Walker S (2015). Fitting linear mixed-effects models using lme4. J Stat Softw.

[36] Kuznetsova A, Brockhoff PB, Christensen RHB (2015). Package ‘lmertest’. R package version.

[37] Bürkner PC (2017). Brms: an R package for Bayesian multilevel models using Stan. J Stat Softw.

[38] Gabry J, Češnovar R. Cmdstanr: R interface to ’CmdStan’. 2021. https://mc-stan.org/cmdstanr

[39] Makowski D, Ben-Shachar MS, Lüdecke S (2019). BayestestR: describing effects and their uncertainty, existence and significance within the Bayesian framework. J Open Source Softw.

[40] Jenkinson M, Beckmann CF, Behrens TEJ, Woolrich MW, Smith SM (2012). FSL. Neuroimage.

[41] Avants BB, Tustison N, Song G, Cook P, Klein A, Gee JC (2011). Advanced normalization tools (ANTS). Neuroimage..

[42] Griffanti L, Douaud G, Bijsterbosch J, Evangelisti S, Alfaro-Almagro F, Glasser MF (2017). Hand classification of fMRI ICA noise components. Neuroimage..

[43] Beckmann CF, Smith SM (2004). Probabilistic independent component analysis for functional magnetic resonance imaging. IEEE Trans Med Imaging.

[44] Pruim RH, Mennes M, Buitelaar JK, Beckmann CF (2015). Evaluation of ICA-AROMA and alternative strategies for motion artifact re- moval in resting state fMRI. Neuroimage..

[45] Breiman L (2001). Random forests. Machine Learning.

[46] Salimi-Khorshidi G, Douaud G, Beckmann CF, Glasser MF, Griffanti L, Smith SM (2014). Automatic denoising of functional MRI data: Combining independent component analysis and hierarchical fusion of classifiers. Neuroimage..

[47] Penny WD, Friston KJ, Ashburner JT, Kiebel SJ, Nichols TE, editors. Statistical parametric mapping: the analysis of functional brain images. Amsterdam: Elsevier; 2011.

[48] McFarquhar M, McKie S, Emsley R, Suckling J, Elliott R, Williams S (2016). Multivariate and repeated measures (MRM): A new toolbox for dependent and multimodal group-level neuroimaging data. Neuroimage..

[49] McFarquhar M (2019). Modeling group-level repeated measurements of neuroimaging data using the univariate general linear model. Front Neurosci.

[50] Baxter M, Murray E (2002). The amygdala and reward. Nat Rev Neurosci.

[51] Pujara MS, Philippi CL, Motzkin JC, Baskaya MK, Koenigs M (2016). Ventromedial prefrontal cortex damage is associated with decreased ventral striatum volume and response to reward. J Neurosci.

[52] Tiedemann LJ, Alink A, Beck J, Büchel C, Brassen S (2020). Valence encoding signals in the human amygdala and the willingness to eat. J Neurosci.

[53] Rolls ET (2023). The orbitofrontal cortex, food reward, body weight and obesity. SCAN.

[54] Wassum KM (2022). Amygdala-cortical collaboration in reward learning and decision making. eLife.

[55] Amaral DC, Price JL (1984). Amygdalo-cortical projections in the monkey (Macaca fascicularis). J Comp Neurol.

[56] Howard JD, Gottfried JA, Tobler PN, Kahnt T (2015). Identity-specific coding of future rewards in the human orbitofrontal cortex. PNAS.

[57] Ard J, Fitch A, Fruh S, Herman L (2021). Weight loss and maintenance related to the mechanism of action of Glucagon-Like Peptide 1 receptor agonists. Adv Ther.

[58] Lu Z, Yeung CK, Lin G, Yew DTW, Andrews PLR, Rudd JA (2017). Centrally located GLP-1 receptors modulate gastric slow waves and cardiovascular function in ferrets consistent with the induction of nausea. Neuropeptides..

[59] Sun L, Kroemer NB, Veldhuizen MG, Babbs AE, de Araujo IE, Gitelman DR (2015). Basolateral amygdala response to food cues in the absence of hunger is associated with weight gain susceptibility. J Neurosci.

[60] Small DM, Zatorre RJ, Dagher A, Evans AC, Jones-Gotman M (2001). Changes in brain activity related to eating chocolate: from pleasure to aversion. Brain.

