# Supplementary material for: A randomized controlled trial investigating the effect of liraglutide on self-reported liking and neural responses to food stimuli in participants with obesity
Source: Int J Obes (Lond). 2023 Aug 25;47(12):1224–31. doi: 10.1038/s41366-023-01370-w (PMC10663148; doi:10.1038/s41366-023-01370-w)
Supplement: Supplementary file 1 — Supplementary information [file 41366_2023_1370_MOESM1_ESM.pdf]

Supplementary information

Eligibility criteria

Participants fulfilling all of the following inclusion criteria were eligible for the study:

1. informed consent as documented by signature
2. age between 18 years and 75 years
3. BMI ≥ 30 kg/m2 and < 45 kg/m2
4. stable body weight (<5% reported change during the previous 3 months)
5. right-handed
6. currently non-smoker (or consuming less than 5 cigarettes per day)

The presence of any one of the following exclusion criteria led to exclusion from the study:

7. Contraindications to Saxenda®, e.g. known hypersensitivity or allergy to Saxenda®,
8. Pregnancy,
9. Renal failure (GFR<30 ml/min)
10. Liver failure (AST>3N and/or ALT>3N)
11. Drugs (e.g., Orlistat, phentermine and topiramate, buproprion and naltrexone taken for the underlying condition, obesity, and any centrally acting medication, glucocorticoides and insulin), were not permitted during the study,
12. Known or suspected non-compliance, drug or alcohol abuse,
13. Inability to follow the procedures of the study (e.g. due to language problems, psychological disorders, dementia),
14. Participation in another study with investigational drug within the 30 days preceding and during the present study,
15. Previous enrolment into the current study,

16. Enrolment of the investigator, his/her family members, employees and other dependent persons,
17. History of any psychiatric diseases, heart failure (NYHA II-IV), type 1 and type 2 diabetes mellitus,
18. History of pancreatitis,
19. Family or personal history of multiple endocrine neoplasia type 2 or familial medullary thyroid carcinoma,
20. Allergies to chocolate, vanilia or strawberry,
21. Deficits of smell and taste,
22. Contraindications for fMRI (e.g. pacemaker or other implanted devices, claustrophobia).

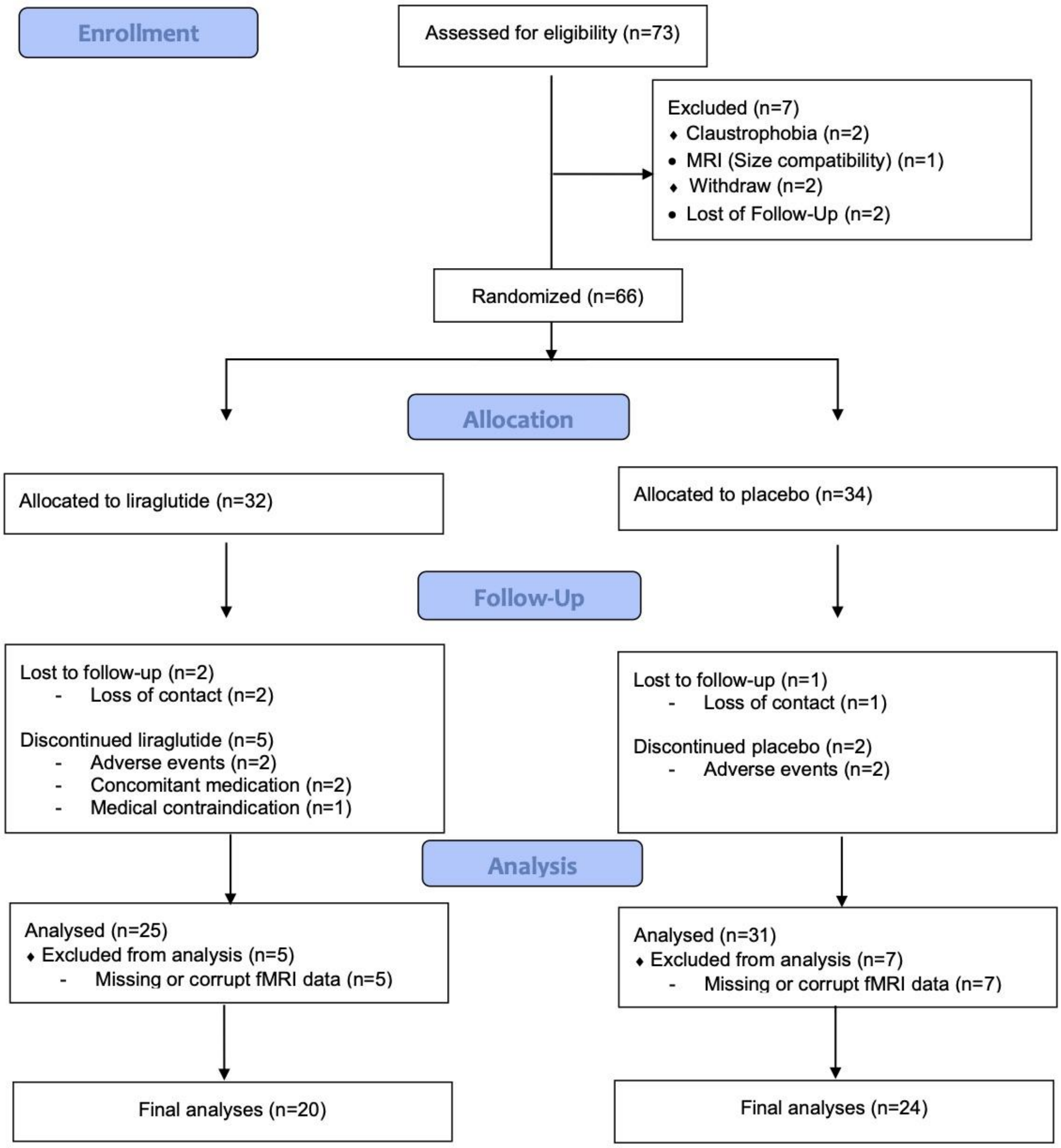

Figure 1. Study flow diagram.

Table 1: Baseline characteristics of the study population

	Placebo average (SD)/N (%)	Liraglutide average (SD)/N (%)	Test
BMI (kg/m^2)	34.88 (2.87)	35.89 (3.01)	t(df=41) = -1.12, p = .268, d = 0.34
Age (years)	40.04 (14.10)	37.40 (11.18)	t(df=41) = 0.67, p = .504, d = 0.21
Body weight (kg)	101.70 (9.77)	102.30 (16.96)	t(df=41) = -0.15, p = .885, d = 0.04
Waist circumference (cm)	109.57 (9.67)	110.00 (12.32)	t(df=41) = -0.13, p = .898, d = 0.04
Sex	Chi-square = 0.11, df = 1, p = .739, Phi = 0.05		
Female (N)	8	6	
Male	15	14	

Table 2: Self-reported levels of hunger, as well as pleasantness, intensity and familiarity ratings for the selected tasteless solution and the milkshake at pretest

	Liraglutide M (SD)	Placebo M (SD)	Test
Hunger level	2.38 (2.93)	1.92 (2.17)	t(df=38) = -0.57, p = 0.571
Pleasantness (tasteless)	5.00 (2.22)	5.19 (1.69)	t(df=38) = 0.30, p = 0.764
Intensity (tasteless)	1.88 (2.29)	2.67 (1.89)	t(df=38) = 1.19, p = 0.242
Familiarity (tasteless)	5.31 (3.46)	5.30 (3.20)	t(df=38) = -0.22, p = 0.825
Pleasantness (milkshake)	7.95 (1.90)	6.62 (2.97)	t(df=37) = -1.65, p = 0.107
Intensity (milkshake)	7.36 (1.88)	7.07 (2.27)	t(df=37) = -0.44, p = 0.666
Familiarity (milkshake)	8.42 (1.93)	7.30 (2.78)	t(df=37) = -1.46, p = 0.149

Table 3: Change in secondary end points from baseline to 16-week follow-up

	Liraglutide M (SD)	Placebo M (SD)	Test
BMI (kg/m2)	-3.19 (1.28)	-0.60 (1.26)	t(df=41) = -6.68, p <.001, d = 2.04
Body weight (kg)	-8.95 (3.65)	-1.78 (3.80)	t(df=41) = -6.28, p<.001, d = 1.92
Waist circumference (cm)	-6.45 (6.31)	-1.30 (8.53)	t(df=41) = -2.22, p = .032, d = 0.68
Systolic blood pressure (mmHg)	4.65 (6.53)	-1.52 (12.28)	t(df=41) = 2.01, p = .051, d = 0.62
Diastolic blood pressure (mmHg)	3.30 (12.08)	2.39 (8.80)	t(df=41) = 0.28, p = .778, d = 0.09
Heart Rate (bpm)	-0.75 (9.60)	-0.96 (14.60)	t(df=41) = 0.05, p = .957, d = 0.02
Fasting Glucoce (mmol/l)	-0.43 (0.59)	0.45 (1.82)	t(df=41) = -2.07, p = .044, d = 0.63
HbA1c (%)	0.03 (1.18)	-0.26 (1.85)	t(df=41) = 0.59, p =.555, d = 0.18
Insuline (mUI/l)	-9.70 (20.47)	1.04 (12.74)	t(df=39) = -2.03, p = .049, d = 0.63
Total cholesterol (mmol/l)	0.14 (0.53)	-0.35 (1.36)	t(df=41) = 1.50, p = .140, d = 0.46
LDL cholesterol (mmol/l)	0.03 (0.57)	-0.46 (1.00)	t(df=41) = 1.96, p = .057, d = 0.60
HDL cholesterol (mmol/l)	0.10 (0.08)	-0.05 (0.32)	t(df=41) = 1.90, p = .064, d = 0.58
Triglycerides (mmol/l)	0.01 (0.52)	0.26 (0.99)	t(df=41) = -1.02, p = .314, d = 0.31

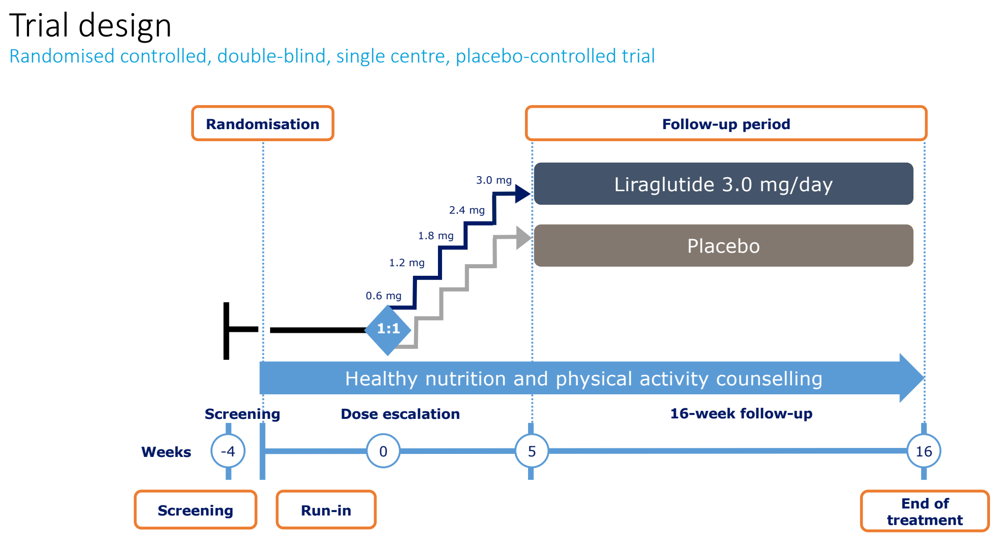

Figure 2. Study design regarding dose escalation.

Table 4. Weight loss results

Frequentist Approach results of the weight model (columns 1 to 5) as well as Bayesian approach results (column 6)

	ß	Standard deviation	Confidence interval of the weight model 2.5%	Confidence interval of the weight model 97.5%	*p* value	Bayesian factor
**Intervention**	0.704	0.105	0.492	0.916	0 .001	> 1000
**Sex**	0.046	0.112	-0.180	0.272	0.680	0.272
**Age**	0.166	0.102	-0.041	0.372	0.113	0.383

Table 5. Liking results

Frequentist Approach results of the liking model (columns 1 to 5) as well as Bayesian approach results (column 6)

	ß	Standard deviation	Confidence interval of the weight model 2.5%	Confidence interval of the weight model 97.5%	*p* value	Bayesian factor
**Stimulus**	-0.409	0.077	-0.560	-0.257	< 0.001	> 1000
**Session**	0.087	0.042	0.004	0.170	0.045	0.289
**Intervention**	0.019	0.072	-0.122	0.159	0.795	0.077
**Satiation**	0.013	0.003	0.007	0.018	< 0.001	85.52
**Stimulus * Session**	-0.073	0.034	-0.141	-0.005	0.042	0.261
**Stimulus * Intervention**	0.024	0.077	-0.128	0.176	0.760	0.091
**Session * Intervention**	-0.004	0.042	-0.087	0.079	0.930	0.045

**Stimulus * Satiation**	-0.007	0.002	-0.010	-0.003	0.002	2.29
**Session * Satiation**	-0.002	0.002	-0.005	0.001	0.190	0.042
**Intervention * Satiation**	0.002	0.003	-0.003	0.008	0.407	0.046
**Stimulus * Session * Intervention**	0.017	0.035	-0.051	0.086	0.617	0.043
**Stimulus * Session * Satiation**	0.001	0.001	<- 0.001	0.003	0.128	0.038
**Stimulus * Intervention * Satiation**	-0.003	0.002	-0.006	0.001	0.174	0.056
**Session * Intervention * Satiation**	<- 0.001	0.002	-0.003	0.003	0.766	0.019
**Stimulus * Session * Intervention * Satiation**	<0.001	< 0.001	-0.001	0.002	0.529	0.012

Additional analysis

As the physiology state (in particular hunger) can in some cases impact both cue-triggered wanting and liking (see reference 10 in the main manuscript), we have run a similar model as the second model reported and entered both hunger and homa_ir as covariates in the statistical model. More specifically, we ran the following model:

perceived_liking_z ~ condition*session*intervention*saturation+ homa_ir+hungry +(condition*session*saturation | id)

This analysis led to the same effects as in the model without the moderator, plus a statistically significant but inconclusive main effect of hunger (see Table 6 and Figure 3). One could expect the inconclusive effect of hunger: what affects the most the liking experience in a paradigm like this one is not the general state of hunger, but rather selective satiation processes that are specific to the food that is consumed during the experiment (i.e., the milkshake). Since our statistical analysis models selective satiation (through the satiation factor), there is little variance left to be captured by general hunger.

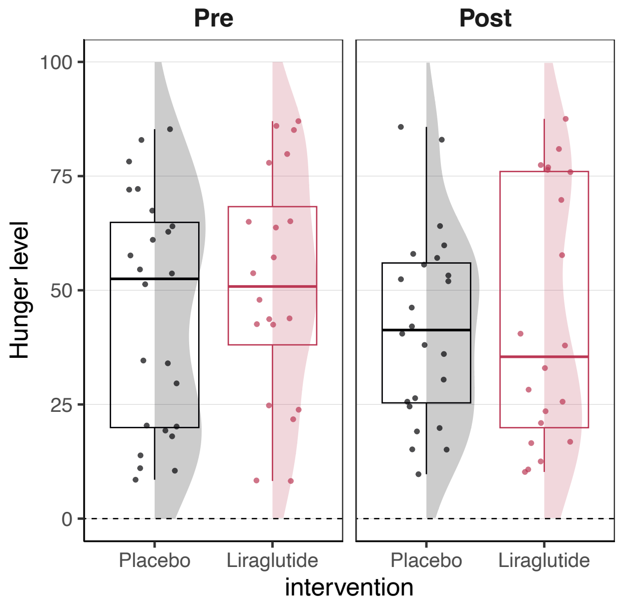

Figure 3. Hunger level before and after the intervention

Table 6. Liking results with hunger and homa_ir as covariates

Frequentist Approach results of the liking model with hunger and homa_ir as covariates (columns 1 to 5) as well as Bayesian approach results (column 6)

	**β**	**Standard deviation**	**Confidence interval of the weight model 2.5%**	**Confidence interval of the weight model 97.5%**	***p* value**	**Bayesian factor**
**Stimulus**	-0.399	0.079	-0.555	-0.244	<0.001	1640
**Session**	0.075	0.423	-0.008	0.158	0.081	0.204
**Intervention**	0.058	0.080	-0.098	0.215	0.468	0.089
**Satiation**	0.013	0.003	0.007	0.018	<0.001	>1000
**Homa_ir**	-0.122	0.089	-0.297	0.052	0.177	0.133
**Hunger**	0.005	0.002	0.001	0.009	0.022	0.007
**Stimulus * Session**	-0.074	0.036	-0.145	-0.003	0.051	0.262
**Stimulus * Intervention**	0.050	0.079	-0.105	0.206	0.530	0.100
**Session * Intervention**	0.009	0.042	-0.073	0.092	0.826	0.044
**Stimulus * Satiation**	-0.007	0.002	-0.011	-0.003	0.001	>1000
**Session * Satiation**	-0.003	0.002	-0.006	>0.001	0.105	0.259
**Intervention * Satiation**	0.003	0.003	-0.002	0.009	0.231	67.820
**Stimulus * Session * Intervention**	0.016	0.036	-0.056	0.087	0.671	0.046
**Stimulus * Session * Satiation**	0.002	0.001	>-0.001	0.003	0.090	0.007

**Stimulus * Intervention * Satiation**	-0.003	0.002	-0.007	0.001	0.176	0.453
**Session * Intervention * Satiation**	>-0.001	0.002	-0.003	0.003	0.855	>0.001
**Stimulus * Session * Intervention * Satiation**	>0.001	>0.001	-0.001	0.002	0.741	>0.001
